# NONINVASIVE INTERVENTIONS IN HEALTHY AND AT-RISK OLDER ADULTS: THE CASE FOR SOCIAL DANCING

**DOI:** 10.1093/geroni/igad104.1263

**Published:** 2023-12-21

**Authors:** Helena Blumen

**Affiliations:** Albert Einstein College of Medicine, Bronx, New York, United States

## Abstract

Physical and cognitive activities can induce neuroplasticity and lead to modest improvements on cognition in healthy and at-risk older adults. Yet, many unanswered questions remain – including what type of physical, cognitive, or combination of physical and cognitive activities, are most beneficial, and to whom (e.g., healthy, or at-risk older adults). This single-blind randomized clinical trial (RCT; NCT03475316) contrasted 6 months of social ballroom dancing with 6 months of treadmill-walking. Twenty-five dementia-at-risk older adults (mean age = 76.45 years; 66% women; 48% White/Caucasian; 24% Black/African American; 20% Hispanic/Latino) were randomized between June 2019 and March 2020. Sixteen participants completed this RCT prior to early study termination due to COVID-19 (8 in each study arm). Dementia-at-risk was defined as a memory impairment screen score of ≥3 to ≤6, and/or and AD-dementia screening interview of ≥1. A composite measure of executive function – generated from digit-symbol-substitution, flanker interference, and walking-while-talking tasks – improved following both social dancing and treadmill-walking. Yet, social dancing generated greater improvements than treadmill-walking on the digit symbol substitution task. No intervention-related differences were observed in brain activation. Less hippocampal atrophy, however, was observed in the social dancing arm than in the treadmill-walking arm. These preliminary findings partly support the hypothesis that social dancing may promote neuroplasticity and benefit cognition to a greater extent than traditional physical activities. This is presumably because social dancing is not only a physical demanding activity, but also a socially and cognitively challenging activity. Yet, these findings need to be confirmed in larger-scale RCTs

